# Thermoanalytical Investigation of the Curing Kinetics of Sodium Silicate as an Inorganic Binder for 3D Sand Printing

**DOI:** 10.3390/ma18030667

**Published:** 2025-02-03

**Authors:** Jakob Glück, Timon Dommaschk, Daniel Jüngst, Jonas Arimont, Andreas Schilling, Martin Fehlbier

**Affiliations:** Department of Foundry Technology, University of Kassel, Emder Straße 1, 34225 Baunatal, Germany

**Keywords:** thermoanalytics, kinetic model, casting, 3D printing, binder jetting, sand core, sodium silicate, additive manufacturing, thermogravimetry, differential calorimetry

## Abstract

This study investigates the development and application of climate friendly processes in the foundry industry, particularly with regard to the use of inorganic binders to reduce emissions and pollution. An inorganic binder system based on water glass, which is used in 3D printing technology for the production of sand molds and core, is being tested and the possibility of determining a kinetic model for the curing kinetics of sodium silicate as an inorganic binder is investigated. The aim is to use a kinetic model to better describe the microwave process currently required in binder jetting for drying the binder and catalyzing the chemical reaction of the binder during curing. For sodium silicate in particular, there is still no scientific knowledge available in this respect, which is why basic investigations based on thermogravimetry or heat flow difference calorimetry must first be carried out. In this way, it should be possible to simulate the drying process in the microwave, which has so far been based on empirical values, in order to maximize the efficiency of this process and also the quality of the components. The results indicate that the weight loss and weight changes depend on the heating rates and that a heating rate of 30 K/min is not sufficient to fully cure the sample at 500 °C. The thermogravimetric analysis (TGA) shows that the fastest weight loss occurs at the beginning of the measurement, indicating a partial pre-curing of the sample before the measurement. From the measurements, an average activation energy of 144.18 kJ/mol could be determined using the Friedman method and 123.36 kJ/mol and 123.31 kJ/mol using the Ozawa–Flynn–Wall and Kissinger–Akahira–Sunose methods, respectively. Measurements of the heat flow at a heating rate of 30 K/min indicate partially exothermic reactions during the curing process.

## 1. Introduction

Stopping climate change caused by anthropogenic CO_2_ emissions has become a central topic in politics and consequently in the economy in recent years. Against this background, new political resolutions have recently been passed to reduce the emission of climate-damaging greenhouse gases. As a result, companies are pushing ahead with the development and application of climate friendly processes and products. In the foundry industry, including iron casting, organic resin binders based on furan or phenol are predominantly used today for the production of cores or sand molds [1]. In particular, this trend is reinforced by the increasing substitution of green sand (bentonite) with core sand [2]. Organic binders offer good strength combined with good disintegration properties, which are essential factors for their use in the foundry industry. On the other hand, emissions during the hardening of the binder due to the use of special gases or the waste disposal of organically bound sands have a negative effect, which represents a considerable environmental burden. The use of alternative inorganic binder systems can considerably reduce emissions, workplace pollution, and environmental pollution in general [1]. The most commonly used inorganic binder is sodium silicate, also called water glass, which provides high dimensional stability, but is considerably more complex to process [1]. In the water glass CO_2_ process, for example, unstable silica (H_2_Si_2_O_5_) is formed when sodium silicate (Na_2_Si_2_O_5_) is gassed with carbonic acid (H_2_CO_3_), resulting in the precipitation of SiO_2_ gels, which are vital for strength. Alternatively, thermal processes can be used for curing.

Nowadays, molding materials are processed in core shooters to produce cores or molded parts. These shoot the sand mixed with binder and additives under pressure into a permanent metal mold and harden it thermally and sometimes using special process gases. In recent years, apart from the already known additive manufacturing technologies in plastics or metal [3,4], 3D sand printing (binder jetting) has become an increasingly industrialized alternative. The inorganic binder system based on sodium silicate has also become available for this manufacturing process. To cure the sand molds, the printing process is followed by a drying process in a microwave oven in order to drive the water separated in the reaction process out of the component in the shortest possible process times. The use of such a process enables prototype molds and small series to be produced quickly and inexpensively with a high degree of design freedom [1,5,6,7]. The relevant drying parameters such as magnetron power, drying time and pause times as a function of the total power of the microwave or the coupling of the microwave energy are currently determined and estimated mainly by experience. This can be remedied by a material model of the molding material in combination with the binary system, which can be used to create a multi-physics simulation in order to determine the component-specific drying times in advance. By using this system, energy can ultimately be saved, productivity increased and component quality improved, since the drying times can be reduced to the reaction-specific minimum. In order to be able to determine the degree of transformation at any time during curing, a kinetic model is required. The determination of the parameters required for this model is the objective of this work.

In the production of water glass, silica sand and alkali carbonate are melted and quenched [8,9]. This product is dissolved in water under high pressure and temperature [8,9]. The resulting sodium silicate molecules dissolved in the water can be linked by the reaction of two silanol groups [10]. This equilibrium reaction results in the formation of a water molecule [10]. The linking of the molecules can lead to the formation of so-called colloids, whose linkage to each other is prevented by a so-called bilayer on the surface of the colloids [11]. During physical curing, this double layer is reduced by the removal of water and the associated change in pH, resulting in the coagulation of the colloids accompanied by curing of the binder [12].

Three-dimensional printed sand molds are produced using the powder-binder process, also known as drop on powder process or binder jetting [13,14]. In this process, a cycle is repeated by first applying a thin layer of sand, spraying the sand with binder at the required locations, and then lowering the build platform [15]. Subsequently, if a water glass binder is used, the molding material must be cured in a microwave oven [16]. In the finishing process, loose sand is removed from the sand mold [15].

## 2. Materials and Methods

**Figure 1:**
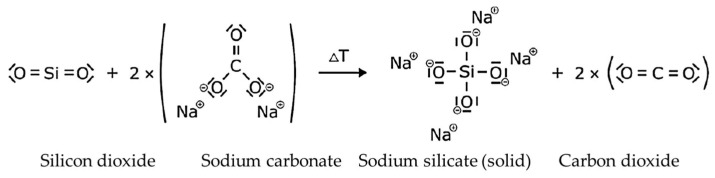
Melting reaction of quartz sand with sodium carbonate.

**Figure 2:**
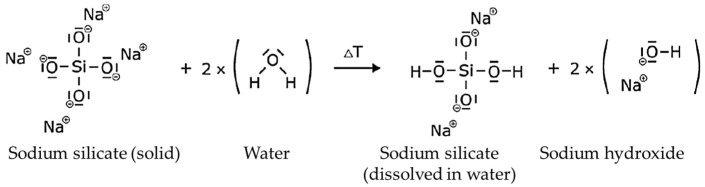
Reactions when dissolving solid sodium silicate in water.

Water glass refers to glass-like solidified melts of alkali silicates and their solutions in water [9]. Water glass is also known as alkali silicate because it consists of alkali oxides and silicon dioxide [8]. The alkali metals used are either sodium, potassium or lithium [8]. Lithium is rarely used because it is more expensive [8]. Sodium is used in 90% of water glass applications [8]. High-purity quartz sand is used as a silicate carrier in the production of water glass [8,9]. It is melted in a furnace with alkali carbonate at 1300–1500 °C to form alkali silicate and cooled abruptly [8,9]. The reaction proceeds as shown in Figure 1 and solid sodium silicate and carbon dioxide are formed. Cooling with water causes the solidified melt to shatter [8]. The resulting so-called lump glass is then dissolved in water at 4–9 bar and 80–180 °C in an autoclave [8,9]. This results in the reaction shown in Figure 2. The products of the reaction are dissolved sodium silicate in water and sodium hydroxide. In another production process for water glass, the hydrothermal process, quartz sand is reacted with sodium hydroxide solution in an autoclave [9]. Finally, certain properties can be adjusted by adding water and alkali lye [8]. The molar ratio can be set with the alkali lye [12]. This is the mass ratio of silicon dioxide to alkali dioxide and is also called the modulus [8]. The solids’ content can also be adjusted [8]. This influences the resulting pore volume during drying [8]. A high molar ratio leads to faster curing speeds and a high strength of the binder [8,9]. As Polzin [9,17] investigated, the molar ratio has no influence on the deformation that occurs during drying. The alkali silicate binders are characterized by their molar ratio, their density and their viscosity. The viscosity of the binder should be as low as possible, as this gives the binder greater fluidity and better wetting of the mold base materials. Its value depends on the molar ratio, the solids’ content, the temperature and the impurities. Finally, the water glass solution is filtered to remove impurities such as alkaline earth, iron and aluminum compounds [12].

**Figure 3:**
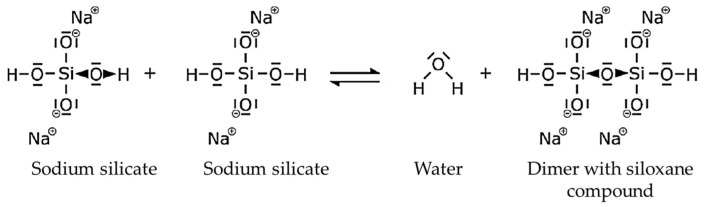
Curing reaction of the sodium silicate binder during physical curing by dehydration.

The solutions of water glasses consist of colloidal ions, silicate anions, water molecules and other cations and anions [9]. As water glass dissolved in water is strongly alkaline, the tetrahedral monosilicate anions initially have protonated silanol groups (Si-OH) [10]. These are very reactive and therefore form a compound with another silanol group [10]. A siloxane group (Si-O-Si) can be formed by a reaction of two molecules with the elimination of water [10]. The reaction described can be seen in Figure 3. Water splits off and the two molecules are chemically bonded [10].

This reaction can be repeated and the molecule increases in size [10]. These binders undergo hardening through various mechanisms including CO_2_ hardening, ester hardening, and thermal hardening. CO_2_ hardening of water glass binders involves a reaction with carbon dioxide, resulting in the formation of a silica gel network that solidifies the binder [11]. This process, known as carbonation, enhances the mechanical properties and stability of the binder, making it ideal for ambient conditions where exposure to CO_2_ is viable [11]. Ester hardening, on the other hand, is achieved through the reaction of water glass with organic esters [8,10]. This reaction leads to the formation of siloxane bonds and the precipitation of silica, which significantly increases the binder’s strength and resistance to water [8]. Thermal hardening involves exposing the water glass to high temperatures, which accelerates the polycondensation of silica, forming a durable, heat-resistant matrix [18].

**Figure 4:**
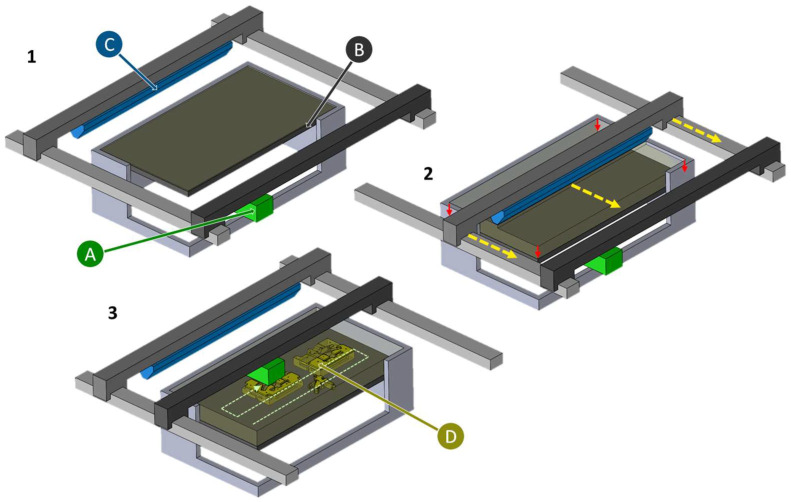
Process steps in the production of a sand casting mold using 3D printing. (1) Printer in initial position. (2) Lowering of the print bed (red arrows) and application of a new sand layer with the recoater (yellow arrows). (3) Meander-shaped printing of the construction area. Repeating the cycle until each layer of sand mold or core is built. With print head (A), print bed (B), recoater (C) and sand molds and cores (D).

The first patent for the application of additive manufacturing in the foundry industry dates back to 1990 [1]. It describes a system that deposits particulate material layer by layer, which is printed with an inkjet print head [1]. This powder-binder process is also known as the drop on powder process or binder jetting [13,14]. In this indirect printing process, the core or sand mold is divided into many layers in a circulation process [15]. The process sequence is described in Figure 4. In the first step, a layer of sand approximately 250 μm thick is applied, which is then printed with binder in the second step [15]. The binder is applied drop by drop to the sand at the locations of the future component and hardens there [15]. Then, in the third step, the job box is lowered by the amount of the layer thickness and the cycle can start again [15]. This creates a core or sand mold, which still needs to be freed from loose sand [15].

According to Utela et al. [7], the binder should be chemically stable and easy to rehydrate and dry slowly before printing. These properties should be reversed after printing. In addition, low viscosity and surface tension are important so that the binder wets the sand grains better and binds the different layers together. The viscosity is determined by the pH value and solid content and can be changed by diluting the binder. The surface tension of the binder can be reduced by adding a liquid with a lower surface tension. The binder jetting process can significantly reduce throughput times and development times [1,5]. Fast printing makes it possible to produce small batches cost effectively [5,6]. In addition, highly complex molds and cores with undercuts that cannot be produced using other methods can be manufactured [1,7]. The dimensional accuracy can be increased by combining several molded parts into one 3D-printed molded part, as this eliminates the stacking tolerances of the previous molded parts [1].

Thermogravimetric analysis (TGA) can be used to investigate the curing of the printed molding material. This allows the weight change in a sample to be studied under a controlled temperature regime and different test-gas atmospheres [19]. The weight changes can be due to volatilization of part of the sample or due to the reaction of the sample with the gas atmosphere [19]. Another way to study the curing of the molding material is to use a dynamic heat flow differential calorimeter (DSC). This can be used to study the heat absorbed or released and the heat flows of materials during controlled temperature variation [20]. Here, a DSC consists of a furnace in which a sample and a reference sample, each with known properties, are placed symmetrically on a disk in crucibles [19]. Under the crucibles are temperature sensors, which can be used to calculate the heat flows of the samples [20]. Calibration is performed with phase changes in pure substances, which also ensure accuracy improvement by determining the thermal resistance between the sample and the temperature sensor and by determining the thermal inertia of the system [20]. In a simultaneous thermal analyzer, TGA measurement and DSC measurement can be performed on the same sample at the same time [21].

For all samples, the devices and materials used are listed in Table 1 and Table 2.

**Figure 5:**
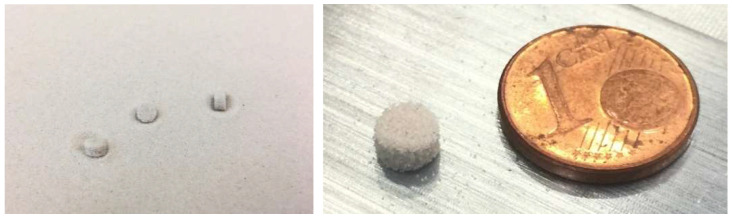
Three-dimensional printed sand samples bound with water glass in sand bed.

In order to be able to guarantee the repeatability of the experimental procedure, the size and weight variations in the sand samples used in the printing process was first investigated on an ExOne SMax-Pro Silicate (ExOne GmbH, Gersthofen, Germany). ExOne FB901 (sodium silicate base, ExOne GmbH, Gersthofen, Germany) was used as binder in the printing process. For this purpose, 20 cylindrical samples with varying diameters were printed and then measured and weighed with high precision. In this way, the printing parameters for the production of cylindrical samples with an almost exact diameter of 5 mm, a height of 3.0 mm and a weight of 67 mg were determined. Figure 5 shows the sand samples in the printing bed. For further sample production for parameter determination, the average deviations of the dimensions and weight were determined.

Furthermore, a Netzsch Jupiter 449 F3 STA instrument (Netzsch-Gerätebau GmbH, Selb, Germany) was calibrated with the melting points of pure metals and measurements were made to investigate the dimensional and weight variation in the printed samples. For all measurements, synthetic air was used as purge gas to create an environment similar to that used in the microwave drying process. In the following, four printed water glass-bonded sand samples were each heated from 30 °C to 500 °C in the STA unit at heating rates of 2 K/min, 8 K/min and 30 K/min, where the selected heating rates were derived on the one hand from the heating rate of the microwave determined in the real process and from graded parameters based on this. It should be noted that a temperature of 500 °C is only reached at the sensor. The sample does not reach this temperature because there is thermal resistance between the sample and the sensor. The samples were examined in aluminum crucibles, since, according to the instrument manufacturer, no reactions occur between them and the samples [22]. In order to be able to evaluate whether and to what extent the drying of the samples occurs prior to measurement, a sample was examined isothermally at 30 °C in the STA device. The measurement results of the melting of an indium sample in an aluminum crucible in the STA device were used to determine the thermal resistance and the time constant for describing the thermal inertia. In a separate test, the weight loss of pure sand and pure binder is investigated. For this purpose, samples of both materials were placed in an oven at 500 °C for four hours and the weight loss was determined. The test is used to determine the proportion by weight of the water glass binder that evaporates and how great the weight loss of the sand is, in order to exclude its influence on the measurement results of the TGA.

To determine the degree of transformation during curing as desired, a kinetic model is required. A kinetic model is a function that describes the quantitative relationship between the reaction rate, the degree of conversion, the temperature and the time [20]. For this purpose, a kinetic equation can be used, which includes the Arrhenius Equation (2) [20], which includes the reaction rate constant k, the Arrhenius factor A, the molar activation energy E_A_, the universal gas constant R, the degree of conversion α, the heating rate β_i_, and temperature T.

(1) $$\frac{d\alpha }{dT}=\frac{A}{\beta_{i}}\cdot e^{\left(-\frac{E_{A}}{R\cdot T}\right)}\cdot \;f(\alpha )$$

(2) $$k\left(T\right)=A\cdot \;e^{\left(-\frac{E_{A}}{R\cdot T}\right)}$$

To solve the kinetic Equation (1), the Arrhenius parameters must be determined as described by Renn et al. [23]. Different model-free methods can be used for this purpose, in which measurement data from experiments with different heating rates are required to determine the Arrhenius parameters. According to this, the differential kinetic function can be determined with the aid of the Arrhenius parameters by comparison with reaction models.

## 3. Results

**Figure 6:**
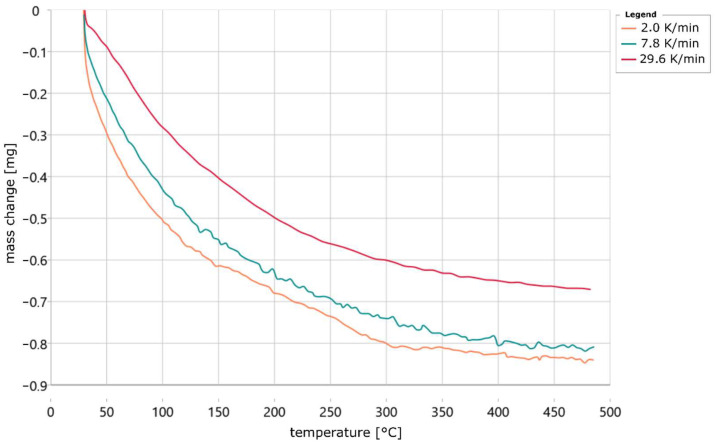
Progress of mass change in 3D-printed waterglass-bonded sand samples at different heating rates plotted depending on temperature.

In the tests on dimensional and weight variation, it was measured that the dimensions of the samples fluctuated by a maximum of 2.63%. In addition, an average weight of 67 mg was measured, which fluctuated by a maximum of 3 mg among the samples, which can be explained by the process at the print head and other influencing parameters such as recoater accuracy, temperature and humidity. The constant dimensions and the small weight fluctuations suggest that the binder content of the samples is approximately constant. The weight loss studies of fresh sand and binder in a normal heat treatment furnace over 4 h and at 500 °C show that the weight of the sand reduces by 0.0497%, which is thus negligible. The pure binder lost 71.49% of its weight and foamed in the process. In the following evaluations of the TGA and DSC results, a thermal resistance of 0.1624 K/mW and a time constant of 6.74 s were used; see description above for indium melt sample. Figure 6 plots the averaged mass change in the four sand samples for each heating rate versus temperature.

**Figure 7:**
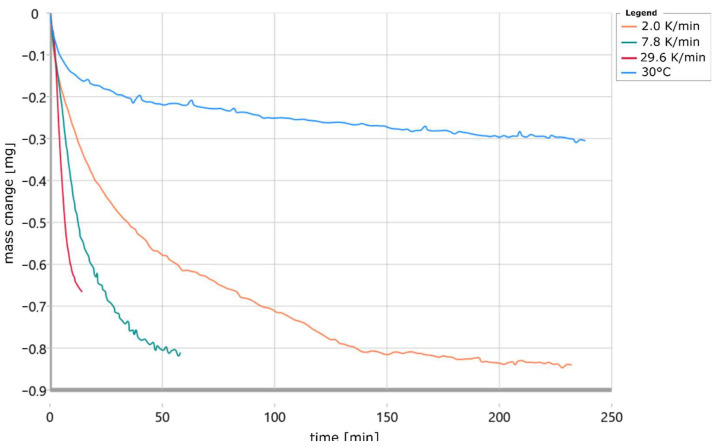
Progress of mass change in 3D-printed water glass-bonded sand specimens at different heating rates plotted depending on time.

The plotted curves were previously corrected in their temperature by the thermal resistance and smoothed. Figure 6 shows that the curves are shifted towards higher temperatures with increasing heating rate; this is typical for a chemical reaction [24,25]. In addition, it can be seen that the samples heated at 30 K/min lost significantly less weight than the other samples at about 480 °C. Furthermore, Köhler’s [17] descriptions that 100% of the water is removed at 400 °C do not apply to the measurements made in this work. This is because it was shown that the samples still lost weight at temperatures above 400 °C. However, it is unclear at what heating rate Köhler examined his samples. However, the lower temperature of 400 °C suggests a very low heating rate. Furthermore, the large initial slope of all curves is striking. This indicates that a large proportion of the specimen has already cured in the approximately 8 min between the end of printing and the start of measurement. The partial curing is therefore due to the time required for the stabilization time of the balance of the STA instrument with 5 min as well as the transport of the sample after the end of printing in a closed container to the STA instrument. In Figure 7, the change in mass of the same specimens is plotted against time.

The measurement curve of the isothermal measurement at 30 °C is also shown here. This shows a similar mass loss at the beginning of the measurement as the other measurements and thus confirms the assumption that part of the binder in the sample has already been cured before the start of the experiment. In addition, a slope of large magnitude is also evident at the end of the mass change curve for the sample heated at 30 K/min. This suggests that the sample is not completely cured at the end of the measurement time. This is because a reaction would be complete when the slope of the mass change curve changes little at the end of the measurement and the mass loss is small. This can be seen well in the case of the specimen heated at 8 K/min, where the mass change decreases successively and this is small at the end of the measurement.

**Figure 8:**
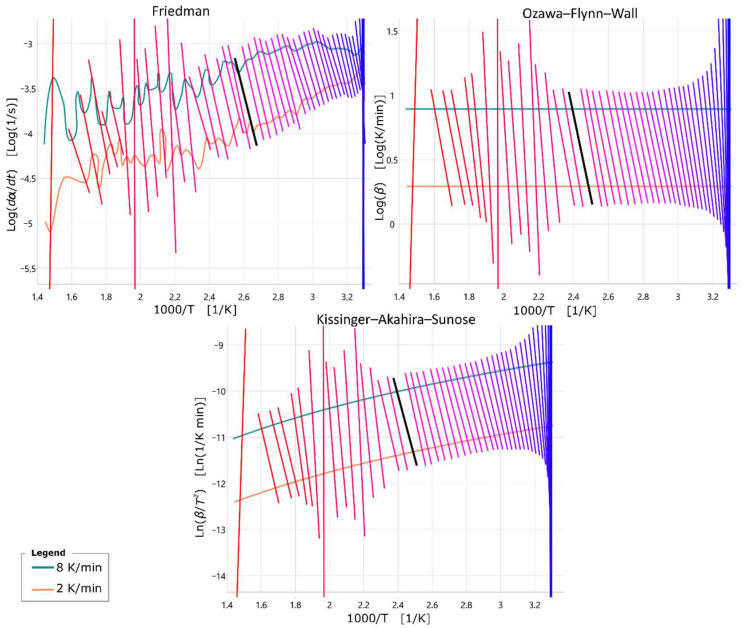
Progress of the straight lines for constant transformation in a calculation with the Friedman, Ozawa–Flynn–Wall and the Kissinger–Akahira–Sunose method for the measurement results at a heating rate of 2 K/min and 8 K/min. Black line indicates a conversion rate of 66%.

To determine the kinetic equation, the conversion degree curves must be determined from the weight loss curves. However, in view of the fact that the previously presented measurements suggest that a proportion of the binder which cannot be identified in these investigations was already chemically converted before the start of the measurement, the conversion degree curves cannot be determined. Moreover, no degree of conversion can be calculated from the curves of the samples heated at 30 K/min, since the measurement results suggest that curing was not complete at the end of the test. Higher temperatures are required for a complete cure, which could not be achieved because the aluminum crucibles limit the maximum test temperature. Although this is not possible, it is still possible to calculate an activation energy that initializes the chemical process of conversion of the binder. Consequently, assuming that the samples heated at 2 K/min and 8 K/min have undergone the same proportion of transformation prior to measurement, the activation energy can be calculated. The transformation progressions were investigated using the Friedman, the Ozawa–Flynn–Wall and the Kissinger–Akahira–Sunose methods [2,26,27]. For this purpose, the conversion curves were plotted in the diagram corresponding to each method, which can be seen in Figure 8.

**Figure 9:**
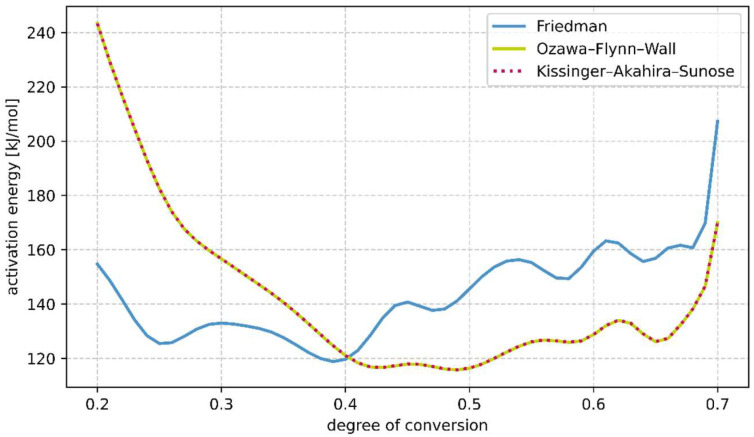
Course of activation energy calculated by model-free analysis methods plotted against the degree of conversion.

In Figure 8, straight lines running through the points of constant conversion have been drawn, whose colour runs from blue to red with increasing conversion. In addition, a black straight line is drawn, which marks a conversion degree of 66%. It can be seen that the Ozawa–Flynn–Wall and Kissinger–Akahira–Sunose methods yield similar straight line curves. The activation energy is related to the slope of the straight lines drawn in Figure 9 and can be calculated for each straight line.

Here, the degree of conversion is plotted in a range from 0.2 to 0.7, since outside this, as can be seen in Figure 9 the activation energy varies greatly. This probably fluctuates because the curing at the beginning of the measurement is not only thermally triggered. In addition, only smaller changes in mass occur at the end of the measurement and the proportion of measurement noise increases. In the results, it can be seen that at the edges of the considered area, the activation energy increases. Moreover, as also observed in Figure 8, the activation energy calculated with the Ozawa–Flynn–Wall and the Kissinger–Akahira–Sunose methods is almost identical. With the Friedman method, in the range from a theoretical conversion of 0.37 to 0.65, taking into account some fuzziness due to the incomplete measurement results described earlier, the average activation energy is 144.18 kJ/mol. Using the Ozawa–Flynn–Wall method, a value of 123.36 kJ/mol can be determined in the same range, and using the Kissinger–Akahira–Sunose method, a value of 123.31 kJ/mol. The so-determined activation energy can now also help in a model-based approach to determine the kinetic model to select the appropriate reaction model.

**Figure 10:**
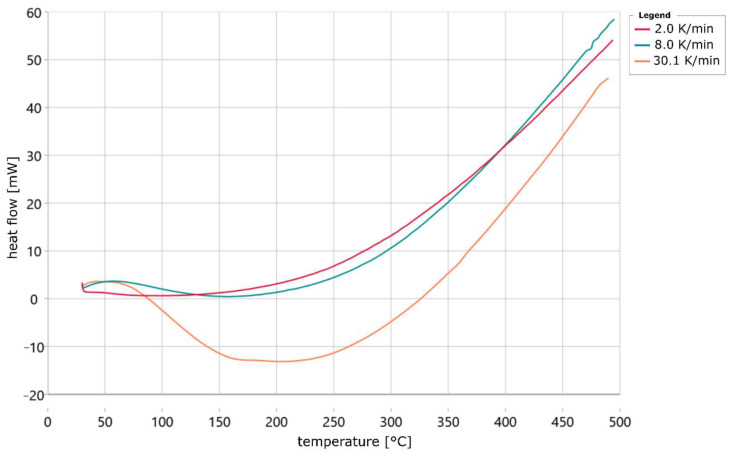
Heat flow plotted versus temperature for continuous heating of water glass 3D-printed sand specimens at different heating rates.

Figure 10 shows the heat flow curves of the samples for the heating rates of 2 K/min, 8 K/min and 30 K/min. The curves are average values from the four tests that were carried out for each heating rate. The results show that there is a maximum in the measurement curve at the heating rates of 8 K/min and 30 K/min at around 50 °C. The measurement curve, recorded at a heating rate of 2 K/min, has no maximum at this point. In addition, the measurement curve of the samples heated at 30 K/min drops sharply after the maximum and reaches negative values for the heat flow. It then rises again until the end of the measurement, but does not reach the measurement curves of the other samples. The measurement curve for the samples that were heated at 8 K/min does not reach negative values. This curve rises from around 160 °C and intersects the curve of the samples heated at 2 K/min at around 400 °C. It can also be seen that the curves of the samples heated at 8 K/min and 30 K/min fall most rapidly in the vicinity of 100 °C. The heat flow that flows into the samples heated at 2 K/min increases continuously from around 80 °C. The heat flows that increase with temperature are probably caused by the quartz sand. This is because its heat capacity increases with temperature [12]. To determine the transformation process and subsequently a kinetic model, baselines would first have to be generated that delimit the heat generated by the reaction. However, this is very difficult because, depending on the heating rate, no clear areas can be defined in which the heat flow is not influenced by the heat of the reaction. In addition, it would be wrong to create a baseline at the start of the measurement, as it was clear from the TG curves that a hardening reaction of the water glass solution was already taking place at the start of the measurement. Therefore, no kinetic model can be created from the heat flow signals. However, it is likely that the curing is an exothermic reaction, as at higher heating rates the filled sample crucible absorbs less heat than the empty crucible. Although this information does not affect the kinetic model, this was not previously known from the existing literature. The largest decrease in the curves measured at a heating rate of 8 and 30 K/min may be related to the boiling point of water, which is 100 °C.

**Figure 11:**
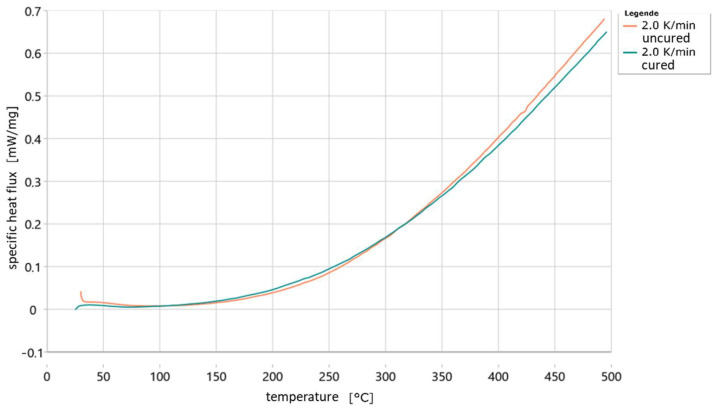
Specific heat flow curves of an already cured and an uncured sample of 3D-printed water glass-bonded sand plotted against temperature.

Figure 11 shows the difference in heat flow for an uncured and an already cured sample at a heating rate of 2 K/min. In contrast to the previous figures, the heat flow here is plotted relative to the weight of the samples. It can be seen that the two curves are very similar. In the range of 100–310 °C, the heat flow absorbed by the already cured sample is greater than that absorbed by the uncured sample. After that, this changes and the uncured sample absorbs a greater heat flow. The similarity of the two curves makes it clear how difficult it is to find a suitable point at which to apply the baseline, as the curves change only slightly due to the heat of the reaction. Further or renewed curing of the already cured sample could be ruled out by the simultaneously recorded TG curve, as no weight loss occurred in this case. Here, the heat flows of the uncured and cured samples differ more clearly. However, it is still not possible to develop a suitable baseline.

## 4. Discussion

Polzin [17] investigated samples of water glass-bonded sands in an STA device, but he used a DTA/TG measuring device in which heat flows are not measured quantitatively. His measurement result is for one of his tests in which a 200 mg sample with a binder content of 9% was heated at a heating rate of 10 K/min. It can be seen that, compared to those measured in the present experiments, the TG curve he measured does not immediately show a negative slope. It first increases and then decreases ever and faster until a temperature of around 115 °C is reached. After that, the rate of weight loss continues to decrease until the weight remains constant at around 500 °C. Compared to the samples measured in this work, the curve measured by Polzin [17] differs significantly in its beginning. In his measurement curve, there is no weight loss at the beginning. In contrast, the measurement results of the experiments carried out in this work showed that the fastest mass loss occurred at the beginning of the measurement. This indicates that the measurement results of Polzin reflect the entire curing process. In addition, at 115 °C, an endothermic peak occurs in the differential thermal analysis curve (DTA curve), paired with a large slope in the TG curve, which is presumably related to the boiling point of water. The differential voltage reaches negative values, which indicates an exothermic reaction. A similar reaction could only be measured in this work at a heating rate of 30 K/min. Here, the sample released heat. As with the curves measured by Polzin [17], the lowest point of the differential voltage and heat flow curve is at approximately 200 °C. These observations reinforce the assumption that the samples analyzed in this work were already partially transformed before the measurement, as the curves measured by Polzin showed a similar course from 150 °C as the measurement results obtained in this work. In addition, Polzin’s [17] results show that the entire transformation can be measured for a 200 mg sample that was produced using a method other than 3D printing. It is also interesting to note that Polzin [17] measured temperatures of 140–150 °C during the microwave drying of water glass-bonded sands. His microwave drying took about 6 min, which corresponds to an average heating rate of over 10 K/min. With the observations made in this work and by Friedman that the measurement curves shift to higher temperatures as the heating rate increases, this means that not all the water has to be removed to achieve high strengths [24].

The results of future research can be improved compared to this work if, at a heating rate of at least 30 K/min, the specimens are heated to above 500 °C so that the entire curing process can be studied. In addition, test specimens with a larger volume-to-surface area ratio than in this work should be used to reduce the drying process of the specimens under isothermal temperature control. One possibility would be a pure TGA measurement where larger crucibles can be used. In addition, the results in this work showed that it is difficult to find a suitable baseline for the DSC measurement results and it is easier to analyze the kinetics of the studied curing reaction with TGA measurements. In addition, it is possible to reduce the stabilization time of the balance of the TGA instrument to the minimum and thus achieve a faster start of measurement. For this, however, the minimum stabilization time must first be determined in a series of tests. For approximate determination of the proportion of the reaction to the change in mass before the start of measurement, a further test may help. By mapping the printing process in the form of manual mixing of the molding sand used with the binder used according to the mass proportions in the printing process, drying carried out isothermally and under constant humidity may reveal the mass change in the range in question here. This would then provide a possibility for approximate determination of the missing measurement results.

## 5. Conclusions

In this study, the possibility of determining a kinetic model for the curing kinetics of sodium silicate as an inorganic binder was investigated. The aim was to use a kinetic model to better describe the microwave process currently required in binder jetting for drying the sodium silicate binder and catalyzing the chemical reaction of the binder during curing. The following findings were made:

- Thermogravimetric (TG) analysis results indicate that weight loss profiles are dependent on the heating rates, with a rate of 30 K/min not sufficing to fully cure the sample at 500 °C. The TG measurement curves show that the fastest weight loss occurs at the beginning of the measurement at all heating rates, suggesting partial pre-curing of the sample prior to measurement, corroborated by isothermal measurement data showing initial weight loss independent of temperature increase. Moreover, the processes appeared to be single-stage, though incomplete descriptions prevent ruling out multi-stage processes. The inability to establish a kinetic model due to incomplete data further complicates this analysis.
- Heating the samples at 2 K/min and 8 K/min, under the assumption of equal conversion prior to measurement, allowed for the determination of the activation energy in a segment of the transformation, yielding average activation energies of 144.18 kJ/mol using the Friedman method, and 123.36 kJ/mol and 123.31 kJ/mol using the Ozawa–Flynn–Wall and Kissinger–Akahira–Sunose methods, respectively. Both latter methods showed similar averages and activation energy profiles.
- Measurements of heat flow at 30 K/min indicated partial exothermic reactions during the curing process. In addition, the heat flow curves of the already cured samples showed how low the heat flow triggered by the curing is, because the curves of the already cured and uncured samples differed only slightly, which makes it almost impossible to develop a suitable baseline. In addition, it would also be wrong to develop a baseline, as the TG measurement results show that the entire transformation is not reproduced. Therefore, no kinetic model can be developed using the heat flow curves.
- To develop a more comprehensive kinetic model, future experiments might explore using larger samples and a pure TG apparatus, with samples heated beyond 500 °C at a minimum of 30 K/min heating rate. Additionally, multi-physics simulation data describing the relationship between strength and degree of transformation would be essential.

## Figures and Tables

**Table 1:** Devices, materials and methods used in three-dimensional printing of sand samples.

3D-printer	ExOne S-Max Pro, ExOne GmbH, Gersthofen, Germany
Sand system	GS 14 RP, Strobel Quarzsand GmbH, Freihung, Germany Mean grain size 0.135 mm
Binder system	ExOne FB901, ExOne GmbH, Gersthofen, Germany sodium silicate base
Curing process	ExOne Microwave, ExOne GmbH, Gersthofen, Germany
Sample size	Cylindrical d = 5 mm, h = 3 mm

**Table 2:** Device and methods used in thermogravimetric analysis (STA) of samples.

STA device	Netzsch Jupiter 449 F3 STA, Netzsch-Gerätebau GmbH, Selb, Germany
Crucible	Aluminum
Testing environment	Synthetic air
Calibration process	Melting points of pure metals

## Data Availability

The original contributions presented in this study are included in the article. Further inquiries can be directed to the corresponding author.

## References

[1] Ramakrishnan R. (2015). 3-D-Drucken Mit Einem Anorganischen Formstoffsystem. Ph.D. Thesis.

[2] Major-Gabryś K. (2019). Environmentally Friendly Foundry Molding and Core Sands. J. Mater. Eng. Perform..

[3] Kohutiar M., Janík R., Krbata M., Bartosova L., Jus M., Timárová Ľ. (2023). Study of the Effect of Pretreatment of 3D Printed PLA Filament Modified by Plasma Discharge and Changes in Its Dynamic-Mechanical Properties. Manuf. Technol..

[4] Wawryniuk Z., Brancewicz-Steinmetz E., Sawicki J. (2024). Revolutionizing transportation: An overview of 3D printing in aviation, automotive, and space industries. Adv. Manuf. Technol..

[5] Holtzer M., Kmita A. (2020). Mold and Core Sands in Metalcasting: Chemistry and Ecology: Sustainable Development.

[6] Findeisen S., Schundmacher S., Woll-Schläger M. (2021). Industrialisierung der Additiven Fertigung.

[7] Utela B., Storti D., Anderson R., Ganter M. (2008). A review of process development steps for new material systems in three dimensional printing (3DP). J. Manuf. Process..

[8] Hopp V. (2019). Einfluss von Aluminium-und Bororthophosphat auf die chemische Härtung von Natron-Wasserglas-gebundenen feuerfesten Rieselmassen. Ph.D. Thesis.

[9] Polzin H. (2012). Anorganische Binder zur Form-und Kernherstellung in der Gießerei.

[10] Wallenhorst C. (2010). Grundlagen zum Verständnis der anorganischen Kernfertigung. GIESSEREI—PRAXIS.

[11] Schuch K. (2014). Steuerung des Aggregationsprozesses in Wässrigen Alkalisilikatsolen Durch Spezielle Gelinitiatoren und Moderate Wärmebehandlung Zum Aufbau Einer Stabilen Silikatbeschichtung. Ph.D. Thesis.

[12] Flemming E., Tilch W. (1993). Formstoffe und Formverfahren.

[13] Gebhardz A., Kessler J., Thurn L. (2016). 3D-Drucken Grundlagen und Anwendungen des Additive Manufacturing (AM).

[14] Almaghariz E.S., Conner B.P., Lenner L., Gullapalli R., Manogharan G.P., Lamoncha B., Fang M. (2016). Quantifying the Role of Part Design Complexity in Using 3D Sand Printing for Molds and Cores. Int. J. Met..

[15] Ramakrishnan R., Griebel B., Volk W., Günther D. (2014). 3D Printing of Inorganic Sand Moulds for Casting Applications. Adv. Mater. Res..

[16] Müller A. (2022). Schrittweise in die grüne Gießerei. Giesserei, 109.

[17] Polzin H. (2000). Untersuchungen zur Mikrowellenverfestigung Von Wasserglasgebundenen Gießereiformstoffen.

[18] Solonenko L., Repyakh S., Uzlov K., Dziubina A., Usenko R. (2020). Sodium Silicate Solute Mass Transferring in Capillary Upon Ultra-High-Frequency Radiation Treatment. Naukovyi Visnyk Natsionalnoho Hirnychoho Universytetu.

[19] Hemminger W.F., Cammenga H. (1989). Methoden der Thermischen Analyse.

[20] Höhne G., Hemminger W., Flammersheim H.-J. (2003). Differential Scanning Calorimetry.

[21] Brown M.E. (1998). Simultaneous Thermal Analysis, Handbook of Thermal Analysis and Calorimetry.

[22] NETZSCH-Gerätebau GmbH Germany Materialverträglichkeit—Probe im Tiegel. https://www.analyzing-testing.netzsch.com.

[23] Renn J., Schlögl R., Schutz B.F. (2017). Non-Isothermal Kinetic Methods.

[24] Friedman H.L. (1964). Kinetics of thermal degradation of char-forming plastics from thermogravimetry. Application to a phenolic plastic. J. Polym. Sci. Part C Polym. Symp..

[25] Mettler: Einfluss der Heizrate: Schmelzen und chemische Reaktion/Influence of the Heating Rate: Melting and Chemical Reaction. https://www.mt.com.

[26] Ozawa T. (1970). Kinetic Analysis of Derivative Curves in Thermal Analysis. J. Therm. Anal..

[27] Lim A.C.R., Chin B.L.F., Jawad Z.A., Hii K.L. (2016). Kinetic Analysis of Rice Husk Pyrolysis Using Kissinger-Akahira-Sunose (KAS) Method. Procedia Eng..

